# Reaching Out in Business

**Published:** 1916-11

**Authors:** 


### EDITORIAL DEPARTMENT

MRS. F. E. DANIEL, Publisher and Managing Editor.

### Reaching Out in Business.

The editor of the TEXAS MEDICAL JOURNAL is in the East in the interest of the publication. Most of the business which makes possible such a publication emanates in the North and East, and it was thought advisable to see personally the men who place the advertising contracts in medical publications and let them know something of the work that is being done in the great Southwest through the TEXAS MEDICAL JOURNAL. However much merit a publication may have, unless it be brought in some way to the attention of the public it will not be appreciated for its full worth. Advertisers need to have their attention called to a publication in a personal way that can hardly be done through the agencies, and it has become the custom of the large publications to have either the publishers or special representatives make special occasional visits to them.

The large growth in the circulation of the TEXAS MEDICAL JOURNAL almost necessitates the adoption of this custom, and so the editor is now away on that business. If the advertisers show that appreciation of the JOURNAL which is manifested by its subscribers, the result will be a substantial increase in patronage that will justify a corresponding increase in the size and the quality of the publication. That is the end which is hoped for, and toward which work is being done.

In the past year the circulation of the TEXAS MEDICAL JOURNAL has extended largely beyond the confines of Texas, and it now has substantial subscription lists in Louisiana, Mississippi, Arkansas, Oklahoma and New Mexico. There is no reason why the list should not be increased until it is recognized as the leading medical journal in the great territory known as the Southwest, if in fact it does not now hold that position.

The leading factor that is making so many friends for it is the list of eminent contributors, who are writing such able articles for it. Probably no other journal in all this section has been so fortunate in securing the assistance of so many learned specialists as contributors. Notice the contents of this number, and you will see that those who have written articles are men who know what they write about and tell it in a professional but understandable way. If energy, determination and persistent effort count for much, this JOURNAL, which is anxious to be regarded as your journal, will be improved from month to month and will become recognized by the entire profession of the Southwest for its helpfulness.

